# Fibroblasts promote the progression of benign prostatic hyperplasia through colony-stimulating factor 1 receptor-mediated RTK signaling in prostatic epithelial cells

**DOI:** 10.1186/s43556-025-00360-w

**Published:** 2025-11-28

**Authors:** Ming Zhan, Ruifeng Yang, Yue Gu, Jun Zhu, Miaomiao Guo, George Pupwe, Xiaohua Huang, Huan Xu, Zhilian Jia, Kyle Takehiro, Chong Liu, Bingyu Li, Yiwei Wang, Yanbo Chen, Xianjin Wang, Qi Chen, Bin Xu

**Affiliations:** 1 Department of Urology, Shanghai Ninth People’s Hospital, Shanghai Jiao Tong University School of Medicine, Shanghai, 200011 China; 2 Department of Systems Biology, Beckman Research Institute, City of Hope, Monrovia, CA 91016 USA; 3 Department of Urology, Fudan University Shanghai Cancer Center, Shanghai, 200032 China; 4 Department of Urology, Putuo People’s Hospital, Tongji University School of Medicine, Shanghai, 200060 China; 5 Department of Molecular Diagnostics & Endocrinology, Shanghai Ninth People’s Hospital, Shanghai Jiao Tong University School of Medicine, Shanghai, 200011 China; 6 Department of Pathology, City of Hope, Duarte, CA 91010 USA; 7 Arcadia High School, Arcadia, CA 91006 USA; 8 Department of Pathology, Mount Sinai West/Morningside Hospitals, New York, NY 10025 USA; 9 Department of Urology, Ruijin Hospital, Shanghai Jiao Tong University School of Medicine, Shanghai, 200025 China

**Keywords:** Benign prostatic hyperplasia, Fibroblasts, Colony-stimulating factor 1 receptor, Receptor tyrosine kinase signaling, Sunitinib

## Abstract

Benign prostatic hyperplasia (BPH) is a prevalent condition characterized by nonmalignant proliferation of epithelial and stromal components in the prostate, frequently resulting in lower urinary tract symptoms in aging men. While receptor tyrosine kinase (RTK) signaling has been implicated in both benign proliferative disorders and malignant tumors, its role in BPH remains insufficiently defined. In this study, transcriptomic analyses of bulk and single-cell RNA sequencing data revealed consistent activation of RTK signaling in prostatic epithelial cells from BPH tissues. Pharmacological inhibition of this pathway using sunitinib suppressed epithelial proliferation in BPH cell lines, organoid models, and an androgen-induced BPH mouse model. Through target screening, colony-stimulating factor 1 receptor (CSF1R) was identified as a central mediator within the RTK signaling cascade. Functional experiments demonstrated that CSF1R promotes epithelial proliferation through activation of the PI3K/AKT/mTOR pathway. Mechanistic studies further showed that fibroblasts, which are expanded in BPH tissues, secrete CSF1 and IL34, both ligands of CSF1R, thereby enhancing downstream signaling and stimulating epithelial growth. Neutralization of these ligands or silencing of CSF1R reversed fibroblast-induced epithelial proliferation and clonogenicity. Clinical observations in patients treated with sunitinib confirmed a significant reduction in prostate volume and improvement in BPH-related urinary symptoms. Collectively, these findings establish a fibroblast/CSF1R/RTK signaling axis that contributes to BPH pathogenesis and support the potential of RTK inhibition as a therapeutic strategy.

**Supplementary Information:**

The online version contains supplementary material available at 10.1186/s43556-025-00360-w.

## Introduction

Benign prostatic hyperplasia (BPH) is a chronic, nonmalignant condition characterized by hyperplasia of prostatic epithelial and stromal components [1]. Its prevalence increases markedly with age, affecting over 50% of men aged 50 years or older, and up to 80% by the age of 80 [1, 2]. Although prostate volume is not the only determinant of lower urinary tract symptoms (LUTS), increased prostate size is significantly associated with a higher risk of acute urinary retention and a greater likelihood of requiring surgical intervention [3]. Current clinical management of BPH primarily relies on pharmacologic therapy, including alpha-adrenergic receptor antagonists, 5-alpha reductase inhibitors, and phosphodiesterase type 5 inhibitors [1]. While these agents alleviate symptoms in many patients, approximately one-quarter of individuals experience limited therapeutic response or progressive disease despite medical treatment [2]. Consequently, more than 20% of men with LUTS ultimately require surgical procedures such as transurethral resection of the prostate (TURP), posing significant risks for elderly patients or those with comorbidities [4]. These limitations underscore the need for improved understanding of the molecular and cellular mechanisms underlying BPH pathogenesis. Identifying novel therapeutic targets may facilitate the development of more effective, mechanism-based interventions and help reduce the burden of disease progression and invasive procedures in aging populations.

Receptor tyrosine kinase (RTK) signaling is a key regulator of fundamental cellular processes, including proliferation, differentiation, and survival [5]. Dysregulated RTK activation is a defining feature of many pathological conditions, particularly fibrotic diseases and malignancies, where it contributes to tissue remodeling and uncontrolled cell growth [6, 7]. In cancer, RTK activation promotes tumor progression through multiple mechanisms, including modulation of the cell cycle, stimulation of angiogenesis, and regulation of the tumor microenvironment [8–10]. RTK inhibitors (RTKis) that target RTKs, particularly vascular endothelial growth factor receptor inhibitors such as sunitinib, sorafenib, and anlotinib, are now established components of targeted cancer therapy [11–13]. Beyond their antitumor effects, these agents can induce structural alterations in non-malignant tissues, raising interest in their potential applications for non-oncologic diseases [7, 14]. In the prostate, RTK signaling has been implicated in the pathogenesis of prostate cancer, partly through regulation of androgen receptor activity by tyrosine phosphorylation [15]. In contrast, the functional significance of RTK pathway activation in BPH remains largely unexplored. A clinical study reported that sunitinib treatment in patients with advanced renal cell carcinoma resulted in measurable reductions in prostate volume and residual urine, suggesting potential therapeutic relevance in BPH [16]. Similarly, imatinib mesylate has been shown to inhibit proliferation of prostatic epithelial cells [17], although the molecular mechanisms involved have not been elucidated. These findings suggest that RTK inhibition may represent a novel therapeutic approach for BPH, underscoring the need for further mechanistic studies.

The tissue microenvironment plays a decisive role in the initiation and progression of disease, shaping cellular behavior through intricate interactions among epithelial, stromal, immune, and vascular compartments. In both malignant and benign conditions, microenvironmental signals regulate proliferation, differentiation, immune surveillance, and therapeutic response [18]. Advances in single-cell RNA sequencing (scRNA-seq) have enabled high-resolution mapping of prostatic cellular ecosystems, allowing detailed characterization of stromal–epithelial, immune–epithelial, and basal–luminal communication [19, 20]. Single-cell studies of the prostate have shown that mesenchymal cells, particularly fibroblasts, actively influence epithelial phenotypes via paracrine signaling. In both mouse and human prostates, fibroblast subtypes display distinct spatial distributions and transcriptional signatures, forming specialized niches that regulate epithelial growth and regeneration [21]. In BPH, fibroblasts adopt altered transcriptional programs, enhance immunoregulatory signaling, and engage in extensive ligand–receptor interactions with epithelial and immune cells, highlighting their role as key mediators of disease-associated remodeling [22]. Findings from prostate cancer further support the central contribution of fibroblasts to disease progression; for example, therapeutic interventions can induce phenotypic switching of cancer-associated fibroblasts, promoting castration resistance through paracrine activation of oncogenic pathways [23]. Collectively, these findings establish fibroblasts as active regulators of the prostate microenvironment, rather than passive structural elements. However, the molecular pathways through which fibroblasts promote epithelial proliferation and contribute to BPH pathogenesis remain incompletely defined, highlighting the need for detailed mechanistic investigation.

In the present study, we examined the contribution of fibroblast–epithelial interactions to the pathogenesis of BPH, focusing on the colony-stimulating factor 1 receptor (CSF1R). Transcriptomic analyses of bulk and single-cell RNA sequencing datasets revealed pronounced activation of RTK pathways in prostatic epithelial cells from BPH tissues. Functional experiments demonstrated that fibroblasts, which are expanded within the BPH microenvironment, secrete the CSF1R ligands CSF1 and IL34, thereby activating PI3K/AKT/mTOR signaling to drive epithelial proliferation and clonogenic growth. Pharmacological inhibition of RTK signaling with sunitinib, as well as CSF1R silencing or ligand neutralization, substantially reduced fibroblast-induced proliferative responses in vitro and suppressed prostate enlargement in an androgen-induced BPH mouse model. Clinical observations further supported these findings, with sunitinib treatment in patients associated with reduced prostate volume and improvement in LUTS. Our findings identify fibroblast-derived CSF1R activation as a central driver of epithelial proliferation in BPH and highlight its potential as a therapeutic target.

## Results

### Activation of RTK signaling in prostatic epithelial cells is associated with the development of BPH

**Fig. 1:**
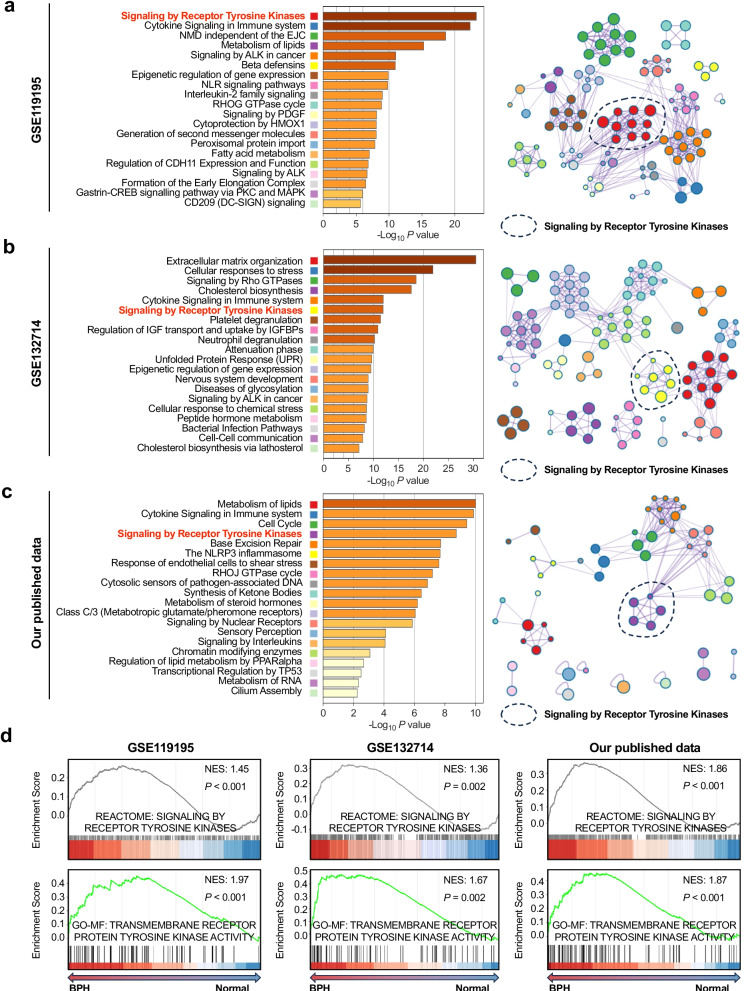
Activation of RTK signaling in prostatic epithelial cells from BPH tissues. **a–c** GO and Reactome pathway enrichment analysis of DEGs in prostatic tissues from the GEO datasets GSE119195 **(a)** and GSE132714 **(b)**, and from our previously published transcriptomic dataset **(c)** revealed significant enrichment of RTK-related pathways in BPH compared with normal prostate tissues. **d** GSEA of the three datasets demonstrated consistent activation of RTK signaling in BPH samples. Normalized enrichment score (NES) and *P* values were shown

To elucidate the signaling pathways implicated in the pathogenesis of BPH, we analyzed two gene expression datasets from the Gene Expression Omnibus (GEO) database (GSE119195: 5 BPH samples and 3 controls; GSE132714: 18 BPH samples and 4 controls) [24, 25]. Gene Ontology (GO) enrichment analysis of differentially expressed genes (DEGs) indicated a consistent and significant enrichment of RTK signaling pathways in both cohorts (Fig. 1a–b). This finding was supported by a previously published transcriptomic dataset from our group [26], which included 6 BPH and 6 normal prostate samples. Similar enrichment of RTK-related pathways was observed (Fig. 1c). Gene set enrichment analysis (GSEA) of all three datasets was consistent with activation of RTK signaling in BPH (Fig. 1d).

Given that bulk RNA sequencing reflects the average expression across heterogeneous cell populations, we next investigated whether RTK pathway activation occurs specifically within prostatic epithelial cells. scRNA-seq data from BPH and normal tissues (GSE172357) were analyzed [22]. Following quality control and batch effect correction, clustering identified six major cell populations: epithelial cells, fibroblasts, endothelial cells, monocytes, T cells, and mast cells (Fig. S1a–1b). We extracted the epithelial cell subset and compared gene expression profiles between BPH and control tissues (Fig. S1c). GO analysis of epithelial cell DEGs showed significant enrichment of RTK-associated pathways in BPH (Fig. S1d). GSEA further demonstrated pronounced upregulation of RTK signaling in epithelial cells from BPH tissues, along with increased expression of key RTK pathway components (Fig. S1e-1f). Together, these bulk and single-cell transcriptomic analyses suggest that activation of RTK signaling in prostatic epithelial cells may be a feature associated with the development of BPH.

### The RTK pathway inhibitor sunitinib suppresses BPH

**Fig. 2:**
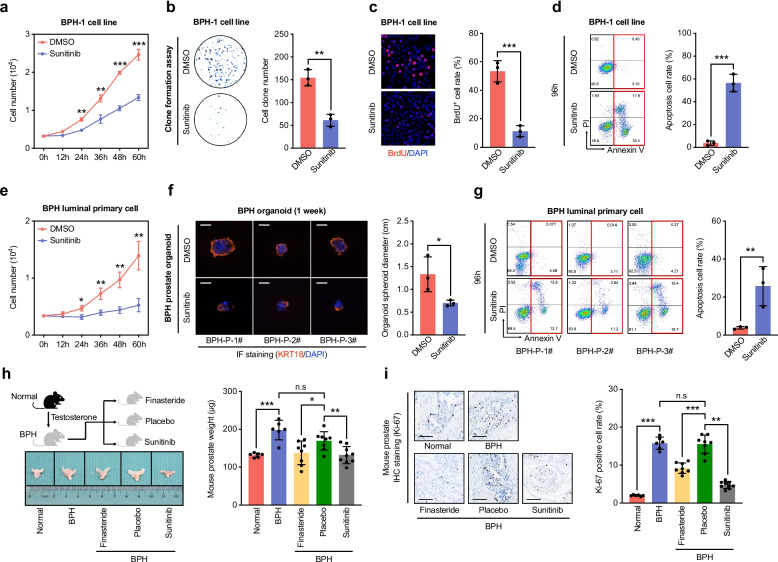
Pharmacological inhibition of RTK signaling with sunitinib suppresses proliferation and induces apoptosis in BPH epithelial cells. **a** Growth curves of BPH-1 cells treated with sunitinib or DMSO for up to 60 h, measured by MTT assay. **b** Representative images and quantification of colony formation in BPH-1 cells under the sunitinib or DMSO treatments. **c** BrdU incorporation assays showing reduced proliferative activity in BPH-1 cells after sunitinib treatment; BrdU-positive cells were visualized by immunofluorescence (BrdU, green; DAPI, blue). **d** Flow cytometric analysis of apoptosis in BPH-1 cells following 96 h treatment with sunitinib, assessed by Annexin V/PI staining. **e** Growth curves of primary luminal epithelial cells isolated from BPH tissues (n = 3 patient-derived cultures) treated with sunitinib or DMSO. **f** Representative images and spheroid diameter quantification of BPH-derived prostate organoids cultured for 1 week with sunitinib or DMSO; luminal epithelial identity confirmed by KRT18 immunofluorescence (green) with DAPI nuclear staining (blue). Scale bars: 1.0 cm. **g** Flow cytometric quantification of apoptosis in BPH-derived primary luminal epithelial cells after 96 h treatment with sunitinib. **h** Comparison of prostate weight in normal or testosterone-induced BPH mice treated with placebo, finasteride, or sunitinib for 8 weeks. **i** Representative IHC staining of Ki-67 and quantification of Ki-67 positive epithelial cells in mouse prostates from the indicated groups. Scale bars: 100 μm. Data are presented as mean ± SD from at least three independent experiments or biological replicates. Statistical significance was determined by Student’s *t*-test; *P < 0.05, **P < 0.01, ***P < 0.001; n.s, not significant

To assess the functional significance of RTK signaling in the development of BPH, we treated both a normal human prostate epithelial cell line (RWPE-1) and a BPH-derived epithelial cell line (BPH-1) with sunitinib, a multi-target RTKi. In vitro assays, including MTT, colony formation, and BrdU incorporation, demonstrated that sunitinib significantly inhibited the proliferation of BPH-1 cells (Fig. 2a–c). Under the same treatment conditions, sunitinib did not affect the proliferation of RWPE-1 cells, as measured by MTT assay (Fig. S2a). Apoptosis analysis further revealed that sunitinib induced apoptosis in BPH-1 cells, whereas RWPE-1 cells remained largely unaffected (Fig. 2d, S2b). To further explore this effect, we isolated luminal epithelial cells from both normal prostate and BPH tissues and evaluated their proliferative response to sunitinib. MTT assays showed a significant reduction in the proliferation of primary luminal cells derived from BPH tissues following sunitinib treatment, while cells from normal tissues showed no significant change (Fig. 2e, S2c). Using these primary cells, we established a 3D prostate organoid culture model. Immunofluorescence staining confirmed expression of the luminal marker KRT18, validating the epithelial identity of the organoids (Fig. 2f). In this system, sunitinib was associated with reduced growth of organoids derived from BPH tissues, whereas organoids from normal prostates were not significantly affected (Fig. 2f, S2d–2e). Apoptosis assays demonstrated that sunitinib induced apoptosis in primary luminal cells isolated from BPH tissues (Fig. 2g).

To evaluate the therapeutic potential of sunitinib in vivo, we established a murine model of BPH by androgen administration. Mice were treated with vehicle, finasteride (as a positive control), or sunitinib. Both finasteride and sunitinib significantly reduced prostate weight and prostate index compared to the vehicle group (Fig. 2h, S2f), without affecting overall body weight (Fig. S2g). Hematoxylin and eosin staining of prostate tissues from the BPH group revealed glandular enlargement and papillary infoldings, which were attenuated by treatment with finasteride or sunitinib (Fig. S2h). Immunohistochemical staining for the proliferation marker Ki-67 showed increased labeling in the BPH group, which was significantly reduced by both finasteride and sunitinib treatment (Fig. 2i). In summary, both in vitro and in vivo evidence is consistent with the notion that pharmacologic inhibition of RTK signaling with sunitinib may attenuate features of BPH.

### CSF1R-mediated activation of RTK signaling promotes proliferation of prostatic epithelial cells

**Fig. 3:**
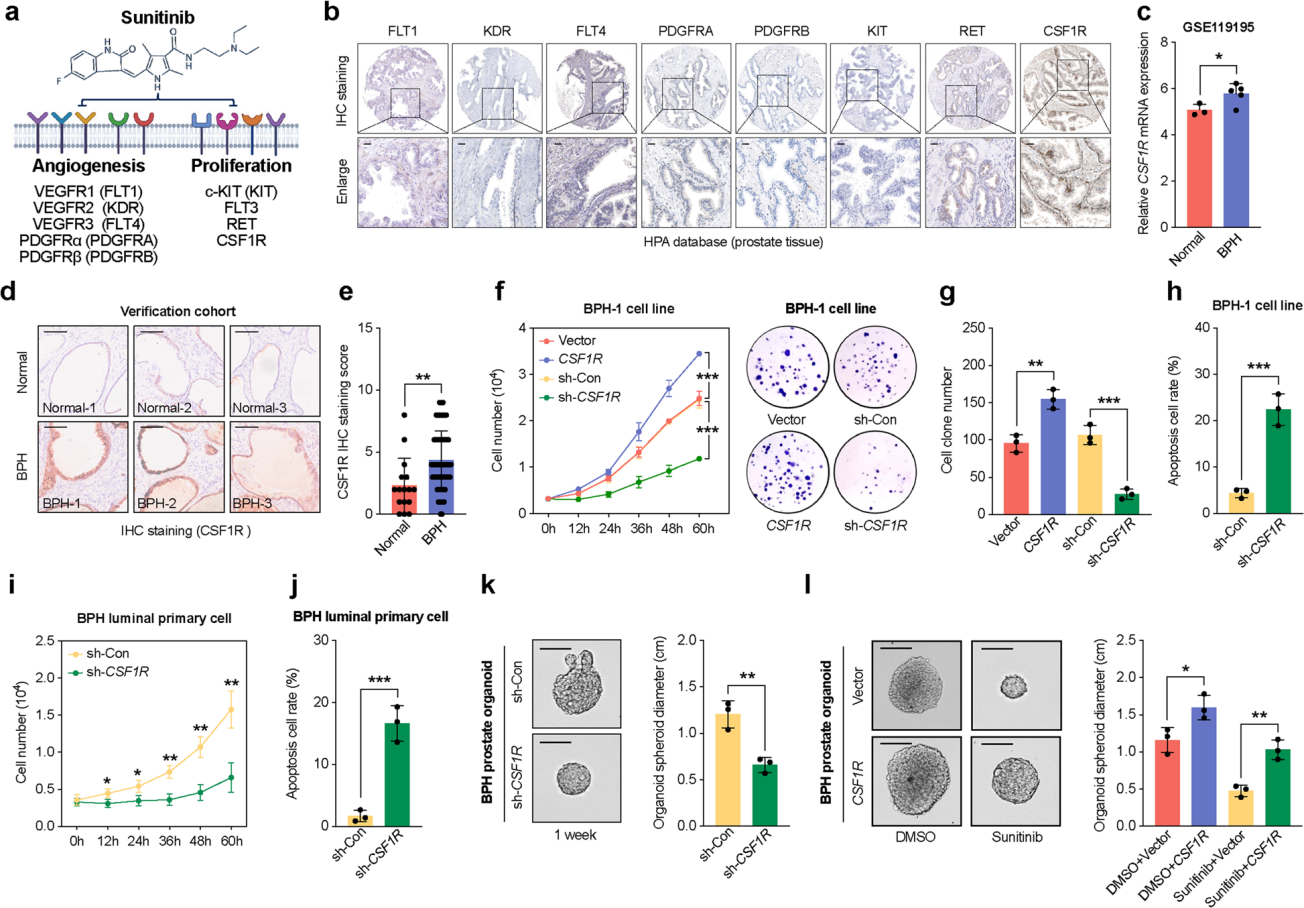
Activation of RTK signaling via CSF1R promotes proliferation of prostatic epithelial cells. **a** Schematic showing the nine established targets of sunitinib, categorized into angiogenesis-related and proliferation-related RTKs. Created with BioRender.com. **b** IHC staining of eight established sunitinib target proteins in prostate tissues from the HPA database. Scale bar: 100 μm. **c** Relative CSF1R mRNA expression in normal and BPH tissues from the GSE119195 dataset. **d–e** Quantification of CSF1R IHC scores in an independent verification cohort (normal, n = 16; BPH, n = 80). Scale bar: 100 μm. **f–g** Growth curves (**f**) and colony formation assays (**g**) of BPH-1 cells transfected with control vector, CSF1R overexpression plasmid, control shRNA, or shRNA targeting CSF1R. **h** Flow cytometric analysis of apoptosis in BPH-1 cells transfected with control shRNA or sh-CSF1R. **i–j** Growth curves (**i**) and apoptosis rates (**j**) in primary luminal epithelial cells isolated from BPH tissues after sh-CSF1R or control shRNA transfection. **k** Representative images and spheroid diameter quantification of BPH-derived prostate organoids transfected with control shRNA or sh-CSF1R. Scale bars: 1.0 cm. **l** Comparison of organoid size in BPH-derived cultures overexpressing CSF1R or vector control under DMSO or sunitinib treatment. Scale bars: 1.0 cm. Data are presented as mean ± SD from at least three independent experiments or biological replicates. Statistical analysis was performed using Student’s *t*-test; *P < 0.05, **P < 0.01, ***P < 0.001

To identify the molecular mediator responsible for RTK pathway activation in prostatic luminal epithelial cells during BPH progression, we first examined the known direct targets of sunitinib. Among the nine established targets, five are primarily involved in angiogenesis (VEGFR1, VEGFR2, VEGFR3, PDGFRα, and PDGFRβ), while the remaining four are linked to cell proliferation (c-KIT, FLT3, RET, and CSF1R) (Fig. 3a). According to immunohistochemical data from the Human Protein Atlas (HPA), immunostaining results were available for eight of the nine sunitinib targets in prostate tissue, except for FLT3. Among these eight proteins, only CSF1R was detected in prostatic luminal epithelial cells (Fig. 3b). Consistently, in our validation cohort comprising 16 normal prostate samples and 80 BPH samples, mRNA expression analysis of the nine sunitinib target genes revealed that only CSF1R was significantly upregulated in BPH tissues, whereas no significant differences were observed for the other eight genes between the two groups (Fig. S3a). This observation led us to hypothesize that CSF1R may act as a mediator of RTK signaling activation in luminal cells.

Analysis of the GEO dataset GSE119195 revealed a significant increase in CSF1R expression in BPH tissues compared to normal controls (Fig. 3c). In our validation cohort, CSF1R upregulation was also observed at the protein level by immunohistochemistry (IHC) (Fig. 3d–e). To clarify the functional role of CSF1R, we conducted both knockdown and overexpression experiments in BPH-1 cells. MTT and colony formation assays showed that CSF1R overexpression enhanced cellular proliferation and clonogenicity, whereas CSF1R knockdown suppressed these phenotypes (Fig. 3f–g). In addition, silencing CSF1R increased apoptosis in BPH-1 cells (Fig. 3h), and pharmacologic blockade with pexidartinib similarly reduced proliferation and increased apoptosis (Supplementary Fig. S3b–3c). Similar findings were observed in primary luminal epithelial cells isolated from BPH tissues, where CSF1R knockdown inhibited proliferation and promoted apoptosis (Fig. 3i–j). In BPH-derived prostate organoid models, CSF1R silencing also led to reduced organoid expansion (Fig. 3k). Finally, overexpression of CSF1R was able to partially rescue the proliferative capacity of both BPH-1 cells and BPH-derived primary luminal cells treated with sunitinib, as shown by MTT assays (Fig. S3d–3e). This reversal effect was corroborated by colony formation and BrdU incorporation assays (Fig. S3f–3 h), and further confirmed in organoid culture models (Fig. 3l). Although CSF1R overexpression alone did not alter apoptosis, it attenuated the pro-apoptotic effect of sunitinib in both BPH-1 cells and BPH-derived primary luminal cells (Fig. S3i–3j). Consistently, sunitinib treatment did not significantly alter CSF1R mRNA or protein expression in either control or CSF1R knockdown BPH-1 cells (Fig. S3k–3l), indicating that its inhibitory effect arises from suppression of CSF1R kinase activity rather than changes in expression. These findings collectively suggest that CSF1R contributes to RTK signaling and promotes proliferation in prostatic luminal epithelial cells, supporting its relevance as a molecular target of sunitinib.

### CSF1 and IL34 promote proliferation of prostatic epithelial cells through CSF1R

**Fig. 4:**
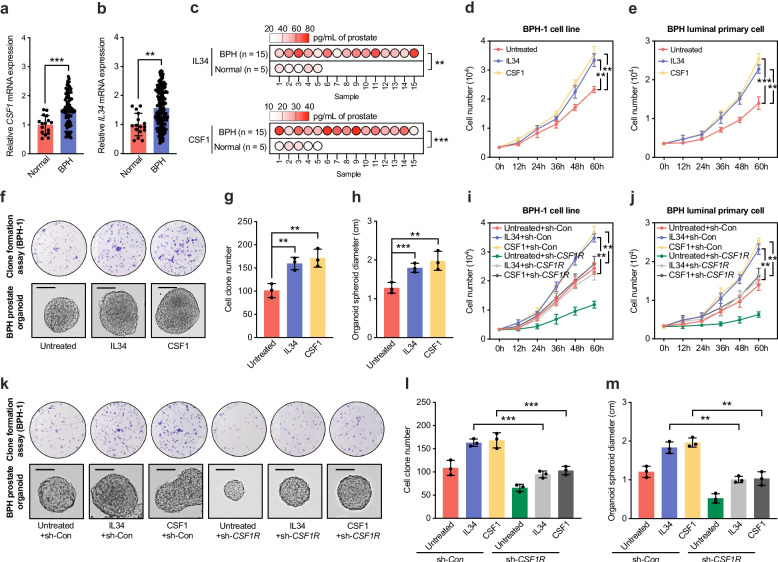
CSF1 and IL34 promote proliferation and clonogenicity of BPH epithelial cells through CSF1R. **a–b** qCR analysis showing elevated CSF1 (**a**) and IL34 (**b**) mRNA levels in BPH tissues compared with normal prostate controls. **c** ELISA quantification of IL34 and CSF1 protein levels in prostate tissue lysates from BPH and normal samples. **d–e** Growth curves of BPH-1 cells (**d**) and primary luminal epithelial cells from BPH tissues (**e**) treated with recombinant CSF1 or IL34, assessed by MTT assay. **f–h** Colony formation assays of BPH-1 cells (**f**, **g**) and prostate organoid growth (**f**, **h**) of BPH-derived epithelial cells after CSF1 or IL34 stimulation. Scale bars: 1.0 cm. **i–j** Growth curves of BPH-1 cells (**i**) and primary BPH luminal epithelial cells (**j**) treated with CSF1 or IL34 after transfection with CSF1R shRNA or control shRNA. **k–m** Colony formation assays (**k, l**) and organoid growth (**k, m**) under the same conditions, demonstrating abrogation of CSF1/IL34-induced proliferation upon CSF1R knockdown. Scale bars: 1.0 cm. Data are presented as mean ± SD from at least three independent experiments or biological replicates. Statistical significance was determined by Student’s *t*-test; *P < 0.05, **P < 0.01, ***P < 0.001; n.s, not significant

CSF1 and IL34 are the two established ligands of CSF1R and can activate the PI3K/AKT/mTOR signaling pathway through receptor binding to regulate cell proliferation and apoptosis [27, 28]. Quantitative PCR analysis showed that the mRNA levels of CSF1 and IL34 were significantly higher in BPH tissues compared to normal controls (Fig. 4a–b). This increase was also confirmed at the protein level using enzyme-linked immunosorbent assay (ELISA) (Fig. 4c). To evaluate the biological effects of these ligands, BPH-1 cells and primary prostatic luminal epithelial cells isolated from BPH tissues were treated with CSF1 or IL34. MTT assays demonstrated a significant increase in cell proliferation following treatment (Fig. 4d–e). This effect was further supported by colony formation assays in BPH-1 cells and by organoid culture experiments using primary luminal cells, both of which showed enhanced clonogenic growth after exposure to CSF1 or IL34 (Fig. 4f–h).

We then investigated whether this proliferative response was mediated by CSF1R. Silencing of CSF1R in both BPH-1 cells and primary luminal cells isolated from BPH tissues attenuated the proliferative effects induced by CSF1 and IL34, as shown by MTT assays (Fig. 4i–j). These results were consistent with colony formation and organoid growth assays, supporting the interpretation that CSF1R is required for the proliferative activity of CSF1 and IL34 (Fig. 4k–m). In addition, stimulation with CSF1 or IL34 led to activation of the PI3K/AKT/mTOR signaling pathway in BPH-1 cells. Silencing CSF1R reduced the basal activity of this pathway and diminished its activation in response to CSF1 and IL34 stimulation (Fig. S4a). Finally, we examined whether sunitinib, a pharmacological inhibitor of CSF1R, could block the actions of these ligands. Both MTT and colony formation assays demonstrated that sunitinib inhibited CSF1- and IL34-induced proliferation and clonogenicity in BPH-1 cells (Fig. S4b–4c). In summary, these findings support that CSF1 and IL34 stimulate the proliferation of prostatic epithelial cells through activation of CSF1R.

### Fibroblasts promote proliferation of prostatic epithelial cells through secretion of CSF1 and IL34

To identify the source of CSF1 and IL34 during BPH progression, we analyzed scRNA-seq data and found that both cytokines were predominantly expressed by fibroblasts within the prostate (Fig. S5a). Fibroblast expansion is a characteristic pathological feature of BPH. To quantify stromal enrichment, we calculated stromal-to-epithelial ratios using three previously described gene expression datasets. All three analyses consistently demonstrated a marked increase in stromal content in BPH tissues compared to normal prostates (Fig. S5b–5d). ELISA results showed that fibroblasts isolated from BPH tissues secreted significantly higher levels of CSF1 and IL34 than prostatic epithelial cells (Fig. S5e–5f).

**Fig. 5:**
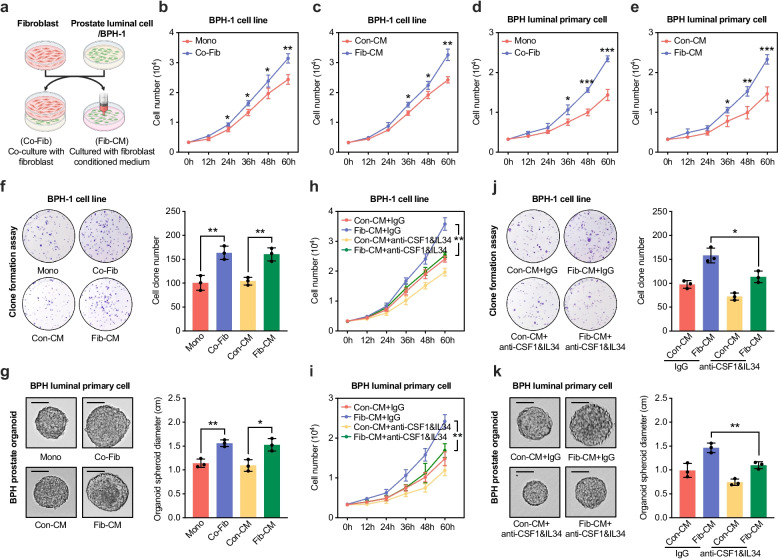
Fibroblasts promote proliferation and clonogenicity of BPH epithelial cells through secretion of CSF1 and IL34. **a** Schematic illustration of the experimental design to investigate fibroblast–epithelial interactions. BPH-1 or primary prostate luminal cells were either co-cultured directly with fibroblasts (Co-Fib) or cultured in fibroblast-conditioned medium (Fib-CM) collected from fibroblast monocultures. Created with BioRender.com. **b–c** Growth curves of BPH-1 cells cultured alone (Mono) or co-cultured with fibroblasts (Co-Fib) (**b**), or cultured with fibroblast-conditioned medium (Fib-CM) or control medium (Con-CM) (**c**), assessed by MTT assay. **d–e** Growth curves of BPH-derived luminal epithelial cells cultured alone (Mono) or co-cultured with fibroblasts (Co-Fib) (**d**), or cultured with control medium (Con-CM) or fibroblast-conditioned medium (Fib-CM) (**e**), assessed by MTT assay. **f–g** Colony formation assays of BPH-1 cells (**f**) and prostate organoid growth from primary BPH luminal epithelial cells (**g**) cultured under Mono, Co-Fib, Con-CM, or Fib-CM conditions. Scale bars: 1.0 cm. **h–i** Growth curves of BPH-1 cells (**h**) and BPH-derived luminal epithelial cells (**i**) treated with Con-CM or Fib-CM in the presence of control IgG or neutralizing antibodies against CSF1 and IL34. **j–k** Colony formation assays of BPH-1 cells (**j**) and prostate organoid growth from primary BPH luminal epithelial cells (**k**) treated with Con-CM or Fib-CM in the presence of control IgG or neutralizing antibodies against CSF1 and IL34. Scale bars: 1.0 cm. Data are presented as mean ± SD from at least three independent experiments. Statistical significance was determined by Student’s *t*-test; *P < 0.05, **P < 0.01, ***P < 0.001

To evaluate whether fibroblast-derived CSF1 and IL34 contribute to epithelial proliferation, we cultured epithelial cells either in direct co-culture with fibroblasts or in fibroblast-conditioned medium (Fig. 5a). MTT assays revealed that both conditions promoted the proliferation of BPH-1 cells and primary luminal epithelial cells derived from BPH tissues (Fig. 5b–e). This proliferative response was further validated by colony formation assays and organoid models, which showed enhanced clonogenic capacity following exposure to either fibroblast co-culture or conditioned medium (Fig. 5f–g). To determine whether these effects were dependent on CSF1 and IL34, we applied neutralizing antibodies to fibroblast-conditioned medium cultures. Simultaneous blockade of both cytokines significantly reduced proliferative and clonogenic responses (Fig. 5h–k). Silencing CSF1R in BPH-1 cells also attenuated the growth-promoting effects of fibroblast-conditioned medium, both in terms of proliferation and colony formation (Fig. S5g–5h). These findings support a model in which fibroblasts promote prostatic epithelial cell proliferation through secretion of CSF1 and IL34, acting via CSF1R signaling.

### Therapeutic potential of the RTK inhibitor sunitinib in the clinical management of BPH

**Fig. 6:**
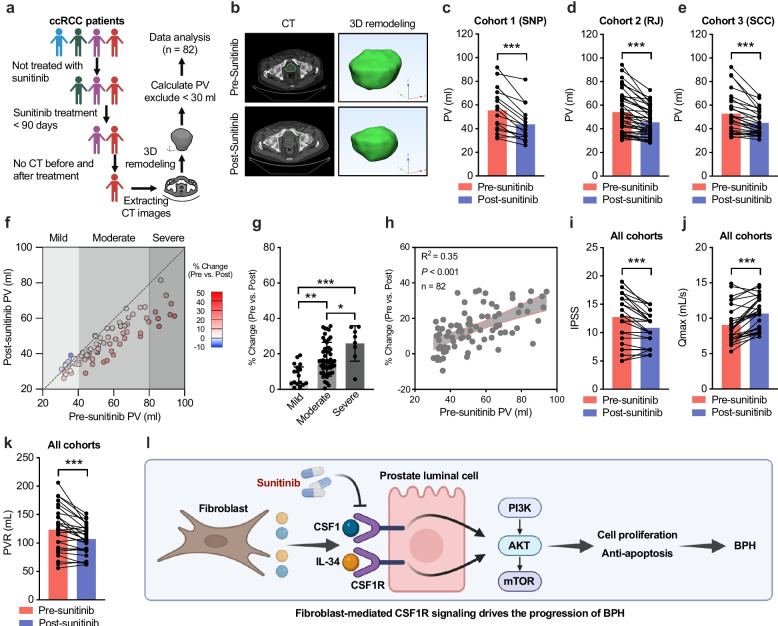
Clinical efficacy of sunitinib in reducing PV and improving LUTS in BPH patients. **a** Workflow for patient selection and PV assessment by 3D reconstruction of CT images. Created with BioRender.com. **b** Representative axial CT and 3D reconstructed images of the prostate from a ccRCC patient before and after sunitinib treatment. **c–e** Individual PV changes before and after sunitinib treatment in Cohort 1 (**c**), Cohort 2 (**d**), and Cohort 3 (**e**). **f-g** Stratification of PV changes according to baseline prostate enlargement severity (mild, moderate, severe). **h** Correlation analysis of baseline PV with the percentage reduction following sunitinib treatment. **i–k** Changes in IPSS (**i**), Qmax (**j**), and PVR (**k**) across all three cohorts before and after sunitinib treatment. **l** Schematic model summarizing the fibroblast–CSF1R–PI3K/AKT/mTOR signaling axis driving epithelial proliferation in BPH and its inhibition by sunitinib. Created with BioRender.com. Data are presented as mean ± SD or as paired pre- and post-treatment values. Statistical significance was determined by paired Student’s *t*-test or Pearson correlation analysis; *P < 0.05, **P < 0.01, ***P < 0.001

To assess the clinical effects of RTK inhibition on the prostate, we evaluated changes in prostate volume and BPH-related symptoms in patients receiving sunitinib. Among three independent cohorts of male patients with clear cell renal cell carcinoma (ccRCC) without concurrent prostate cancer, a total of 673 individuals had received sunitinib for more than 90 days. Of these, 139 had available computed tomography (CT) scans that included the pelvic region both before and after treatment. Prostate volumes were quantified through 3D reconstruction of CT images, and patients with baseline volumes less than 30 mL were excluded. Eighty-two patients met the inclusion criteria and were enrolled for analysis (Cohort 1: SNP, n = 16; Cohort 2: RJ, n = 39; Cohort 3: SCC, n = 27) (Fig. 6a). Across all three cohorts, sunitinib treatment was associated with a significant reduction in prostate volume (Fig. 6b–e). Stratification by baseline prostate size showed that patients with severe prostatic enlargement experienced the greatest reduction in volume (Fig. 6f–g). Moreover, the percentage decrease in prostate size was positively correlated with the initial volume (Fig. 6h). We further examined changes in BPH-related clinical parameters. Following treatment, patients exhibited significant improvements, including reductions in the International Prostate Symptom Score (IPSS) and post-void residual urine volume (PVR), along with an increase in maximum urinary flow rate (Qmax), indicating symptomatic benefit (Fig. 6i-k). Collectively, these findings are consistent with sunitinib reducing prostate size and improving LUTS, supporting its potential as a therapeutic strategy for BPH.

## Discussion

BPH is a prevalent age-related disorder characterized by nodular hyperplasia of the prostate transition zone, leading to progressive LUTS that substantially impair quality of life in older men [29]. Although pharmacological treatments such as α-blockers and 5α-reductase inhibitors can alleviate symptoms in many cases, a considerable proportion of patients respond poorly or ultimately require surgical intervention [30]. While surgical procedures such as TURP can effectively relieve obstruction, a substantial proportion of elderly patients are not suitable surgical candidates due to comorbidities or perioperative risks [31], emphasizing the need for alternative medical approaches. In this study, our data indicate that fibroblast-driven activation of CSF1R may act as a central driver of epithelial proliferation in BPH. Bulk and single-cell RNA sequencing analyses revealed marked enrichment of RTK-related pathways in epithelial cells from BPH tissues. Functional studies suggest that stromal fibroblasts secrete CSF1 and IL34, thereby activating PI3K/AKT/mTOR signaling and promoting epithelial proliferation and clonogenic growth. Inhibition of this pathway with sunitinib, CSF1R silencing, or ligand neutralization was associated with reduced fibroblast-induced proliferation in vitro and attenuated prostate enlargement in an androgen-induced BPH mouse model (Fig. 6l). These results suggest a stromal–epithelial signaling axis in BPH and support CSF1R as a potential therapeutic target.

RTK signaling is a central regulator of cellular proliferation, differentiation, and survival, and its precise control is essential for maintaining tissue homeostasis [5]. Aberrant RTK activation is a frequent feature of human disease, particularly in cancer, where it promotes tumor growth, invasion, and therapeutic resistance through diverse downstream pathways [6, 32]. The clinical relevance of this pathway is reflected in the development of RTK inhibitors such as sunitinib and imatinib, which have been widely adopted in oncology and have improved outcomes in malignancies including renal cell carcinoma, gastrointestinal stromal tumors, and chronic myeloid leukemia [33, 34]. In the prostate, dysregulated RTK signaling has been implicated in the initiation and progression of prostate cancer, partly through modulation of androgen receptor activity via tyrosine phosphorylation [35]. By contrast, the functional significance of RTK pathway activation in BPH has remained unclear. In this study, we characterized RTK signaling activity in BPH, which appears elevated in epithelial compartments. Bulk RNA sequencing analysis revealed marked enrichment of RTK-related pathways in BPH tissues compared with normal controls. scRNA-seq further localized this activation predominantly to epithelial cell populations within the hyperplastic prostate. Functional studies indicated that pharmacological inhibition of RTK signaling with sunitinib is associated with reduced epithelial proliferation in BPH cell lines, primary epithelial cultures, and patient-derived organoids. In an androgen-induced BPH mouse model, sunitinib treatment was associated with a reduction in prostate size, consistent with the in vitro observations. These findings suggest that RTK pathway activation contributes to epithelial expansion in BPH and support the exploration of RTK-targeted strategies as a potential therapeutic approach.

RTKis act primarily by blocking receptor activation at the cell membrane, thereby suppressing downstream signaling cascades [11]. Sunitinib, a multi-target RTKi, inhibits nine membrane-associated receptors [34], but analysis of the Human Protein Atlas indicated that only CSF1R is expressed in prostatic epithelial cells. CSF1R, a class III RTK, is well known for regulating the survival, proliferation, and differentiation of mononuclear phagocytes and has been implicated in immune modulation and tumor progression through macrophage recruitment and polarization [36]. In prostate cancer, CSF1R activation enhances tumor-associated macrophage infiltration and contributes to resistance under androgen deprivation therapy, underscoring its potential as a therapeutic target in malignancy [37]. However, its functional role in BPH has not been defined. Our data suggest that CSF1R contributes to epithelial proliferation in BPH and that sunitinib’s growth-suppressive effect may be at least partly mediated by CSF1R inhibition. CSF1 and IL34, the principal ligands for CSF1R, were significantly upregulated in BPH tissues compared with normal prostate and stimulated epithelial proliferation via CSF1R activation.

Increasing evidence underscores the pivotal role of the tissue microenvironment in disease development. In malignancies and fibrotic disorders, fibroblasts act as active modulators of tissue microenvironment remodeling, influencing cell proliferation, immune regulation, and therapy resistance through diverse paracrine mechanisms [38, 39]. Comparable processes are implicated in BPH, where stromal–epithelial interactions contribute to abnormal epithelial expansion. Spatial transcriptomic and functional studies have identified fibroblasts in BPH stroma as prominent sources of growth factors, including IGF1 and CXCL13, that stimulate epithelial proliferation and morphogenesis [40]. Other studies have shown that immune–stromal interactions, such as M2 macrophage-derived IL-4 signaling, promote a myofibroblast phenotype that drives stromal hyperplasia, while aberrant TGF-β/ROCK1 activation recruits mesenchymal stem cells and fosters fibroblast differentiation [41, 42]. These findings collectively highlight fibroblasts as central mediators of BPH-associated tissue remodeling. In this study, we show that fibroblasts enhance prostatic epithelial proliferation through CSF1R-dependent activation of PI3K/AKT/mTOR signaling. Furthermore, pharmacological blockade of this pathway with sunitinib suppressed fibroblast-induced epithelial proliferation in vitro and reduced prostate enlargement in an androgen-induced BPH mouse model, supporting the potential of CSF1R-targeted strategies for BPH treatment.

Finasteride, which directly inhibits the survival and proliferation of prostatic epithelial cells, remains the first-line pharmacological therapy for BPH [43]. However, a proportion of patients show limited response or develop tolerance, underscoring the need for alternative or targeted treatments. Several molecularly targeted strategies have been investigated, including mTOR inhibitors, which have been associated with prostate volume reduction in specific patient subgroups [25], and Rho kinase inhibitors, which can attenuate hyperplasia, fibrosis, and epithelial–mesenchymal transition in preclinical models [44]. In contrast, studies of RTKis in BPH are scarce. In this study, pooled analyses from three independent patient cohorts indicated that sunitinib treatment significantly reduced prostate volume, in agreement with observations by Takashi et al. in a Japanese cohort [16]. Although that smaller study did not detect a significant improvement in symptom scores, our combined analysis showed that sunitinib not only reduced prostate size but also improved key clinical parameters, including IPSS, Qmax, and PVR, with the greatest volume reduction in patients with larger baseline glands. While we did not evaluate whether sunitinib or other TKIs directly affect 5-α-reductase, potential crosstalk between RTK signaling and the androgen/AR axis could indirectly influence 5-α-reductase and AR activity. Accordingly, future work should test TKI plus 5-α-reductase inhibitor combinations to determine whether complementary mechanisms yield additive or synergistic benefit, particularly in finasteride-refractory BPH.

This study has several limitations. First, CSF1R is a key regulator of inflammation and immune responses [45], both of which are integral to BPH pathogenesis. Thus, the observed therapeutic effects of sunitinib may partly reflect modulation of the prostatic inflammatory and immune microenvironment, a possibility that requires further clarification. Second, we did not mechanistically dissect the interplay between AR signaling and the CSF1R-driven RTK–PI3K–AKT pathway. Our data indicate CSF1R activity across AR-positive/negative models, suggesting a parallel, microenvironment-initiated mechanism that warrants dedicated study. Third, the absence of a prostate epithelial cell–specific CSF1R knockout model limits our ability to provide direct in vivo evidence for the epithelial contribution of CSF1R to BPH progression. Finally, the clinical cohorts analyzed were composed primarily of patients with renal cell carcinoma and concurrent prostate enlargement, which may introduce confounding variables. Prospective studies in patients with primary BPH, free of concomitant malignancy, will be important to confirm these findings and establish their broader clinical applicability.

## Conclusion

This study suggests a mechanism whereby fibroblast-derived activation of CSF1R promotes prostatic epithelial proliferation in BPH. Fibroblasts enriched within the hyperplastic prostatic stroma secrete CSF1 and IL34, which engage CSF1R on epithelial cells, activating PI3K/AKT/mTOR signaling and thereby enhancing epithelial growth and clonogenic potential. Disruption of this axis via sunitinib, CSF1R silencing, or ligand neutralization was associated with reduced fibroblast-induced epithelial proliferation in vitro and attenuated prostate enlargement in an androgen-induced BPH mouse model. In patients receiving sunitinib, measurable reductions in prostate volume and improvements in lower urinary tract symptoms were observed, supporting the potential translational relevance of these findings. Although these results support CSF1R inhibition as a promising, mechanism-based therapeutic approach for BPH, the clinical use of sunitinib should be considered with caution, carefully weighing potential adverse effects against anticipated therapeutic benefits.

## Materials and methods

### Sample and clinical data collection

A total of 80 BPH tissue samples and 16 normal prostate tissue samples were included in this study. BPH specimens were obtained from patients undergoing TURP, whereas normal prostate tissues were collected exclusively from patients undergoing radical cystectomy. Exclusion criteria for BPH specimens included histologically confirmed carcinoma within the transition zone, a diagnosis of hypogonadism, and a prostate volume < 30 mL. All research-designated prostate tissues were harvested from the transition zone during surgery. Portions of each specimen were snap-frozen in liquid nitrogen for RNA extraction, while other portions were processed into frozen and paraffin-embedded sections for histopathological assessment. Selected fresh specimens were additionally used for primary cell isolation and culture, as well as for the establishment of prostate organoids. The cohort evaluating the effects of sunitinib on prostate volume was derived from three independent medical centers: Shanghai Ninth People’s Hospital affiliated to Shanghai Jiao Tong University School of Medicine (SNP), Ruijin Hospital affiliated to Shanghai Jiao Tong University School of Medicine (RJ), and Shanghai Cancer Center affiliated to Fudan University (SCC). Eligible patients were those with a confirmed diagnosis of ccRCC who had received sunitinib for more than 90 days without concurrent administration of BPH-related medications such as finasteride or α-adrenergic receptor antagonists. Patients without available pelvic CT imaging both before and after sunitinib treatment were excluded. Prostate volumes were measured from CT data using 3D reconstruction. Patients with a baseline prostate volume < 30 mL were excluded from analysis. A total of 82 patients met the inclusion criteria: SNP, n = 16; RJ, n = 39; SCC, n = 27. The clinical characteristics of the ccRCC patients from the three cohorts are summarized in Supplementary Table 1. This study was approved by the Ethics Committee of Shanghai Ninth People’s Hospital, Ruijin Hospital, and Shanghai Cancer Center. All procedures were performed in accordance with the approved protocols. Written informed consent was obtained from all participants, and clinical data were handled in compliance with confidentiality requirements.

### Prostate volume measurement via CT-based 3D reconstruction

All participants underwent pelvic CT with 1-mm slice thickness; contrast was administered when clinically indicated. Digital Imaging and Communications in Medicine data were imported into Mimics 21.0 (Materialise, Belgium) for segmentation and 3D modeling. The prostate was manually contoured on each axial slice and reviewed in sagittal and coronal planes, including the entire gland from apex to base but excluding the seminal vesicles, periprostatic tissues, and bladder neck. Following prior contouring guidelines, the inferior boundary was not extended below the level of the obturator foramen in ambiguous cases. A 3D surface model was generated in Mimics and the prostate volume automatically calculated in 3-matic (Materialise) using mesh-based volumetry. All models were checked for mesh integrity, and a random 20% subset was re-contoured by a second reader to assess inter-observer reproducibility.

### Prostate organoid culture and validation

Tissues from benign prostatic hyperplasia and normal prostate were finely minced and digested overnight with 5 mg/mL collagenase II (Thermo Fisher Scientific). Following digestion, the cell suspension was centrifuged, resuspended in PBS, and subjected to differential adhesion to enrich epithelial cells by removing rapidly adherent stromal cells. The remaining suspension was dissociated into single cells using TrypLE (Life Technologies, USA) containing 10 μM Y-27632 (Sigma-Aldrich). After filtration through a 40 μm cell strainer, 20,000 single cells were embedded in 40 μL Matrigel (Corning, USA) and cultured in DMEM/F12 medium supplemented with 5 ng/mL EGF (PeproTech, USA), 10% R-spondin1–conditioned medium, 100 ng/mL recombinant Noggin (PeproTech), 500 nM A83-01 (Tocris Bioscience, USA), 10 ng/mL FGF10 (PeproTech), 5 ng/mL FGF2 (PeproTech), 1 mM prostaglandin E2 (Tocris), 10 μM SB202190 (Sigma-Aldrich, USA), 10 mM nicotinamide (Sigma-Aldrich), and 1 nM dihydrotestosterone (Sigma-Aldrich). Organoid size was evaluated by phase-contrast microscopy after 1 and 2 weeks of culture. Prostate epithelial identity was confirmed by immunofluorescence staining for the epithelial marker KRT18 (anti- KRT18; Cell Signaling Technology, #4548; dilution 1:800).

### Fibroblast co-culture and conditioned medium treatment

Primary fibroblasts were isolated from BPH tissues by collagenase digestion and cultured in DMEM supplemented with 10% FBS. For indirect co-culture, fibroblasts were seeded in the lower chamber of a Transwell system (0.4 μm pore size), and BPH-1 or primary prostate luminal epithelial cells were seeded in the upper chamber. Co-cultures were maintained for 48 h before subsequent assays. Fibroblasts derived from BPH tissues were cultured to 80% confluence, and the medium was replaced with serum-free DMEM for 48 h. The collected fibroblast-conditioned medium was centrifuged to remove cell debris and applied to BPH-1 or primary prostate luminal epithelial cells for indicated treatment durations.

### Plasmid construction and lentiviral infection

CSF1R shRNA (sh-*CSF1R*) and a non-targeting control (sh-Con) were designed and synthesized by the Department of Biochemistry and Molecular Cell Biology, Shanghai Jiao Tong University School of Medicine. The open reading frame of CSF1R was inserted into the pLVX-puro vector. For lentiviral production, HEK293T cells at 80–90% confluence in 100 mm dishes were co-transfected with 3.5 μg of the shRNA or overexpression plasmid, 2.25 μg of psPAX2, and 4.25 μg of pMD2.G using 30 μL of polyethyleneimine. After 6 h, the transfection medium was replaced, and viral supernatants were collected 72 h later. BPH-1 cells and primary prostate luminal cells were infected with lentivirus for 24 h, followed by selection with 2.5 μg/mL puromycin to generate stable cell lines. The shRNA sequences are listed in Supplementary Table 2.

### Animal experiments

Male ICR mice (6–8 weeks old) were acclimated for 2 weeks prior to experimentation. A total of 37 mice were randomly allocated to either a normal control group (n = 6) or a BPH induction group (n = 31). For BPH induction, testosterone propionate was dissolved in olive oil and administered by daily subcutaneous injection at 7.5 mg/kg for 14 consecutive days; control mice received equivalent volumes of olive oil. After 14 days, six mice from the BPH group received no further intervention and were observed until the end of the experiment to serve as the model baseline. The remaining mice were assigned to one of three treatment groups: vehicle control (n = 8; daily oral gavage of 10% PEG400), finasteride (n = 8; 1 mg/kg/day by oral gavage), or sunitinib (n = 9; 5 mg/kg/day by oral gavage). After 8 weeks of treatment, mice were euthanized by cervical dislocation. Prostate tissues were dissected, weighed, and fixed in formaldehyde. The prostate index (PI) was calculated as prostate weight divided by body weight. All procedures were performed in accordance with institutional guidelines and were approved by the Animal Experimentation Ethics Committee of Shanghai Ninth People’s Hospital, Shanghai Jiao Tong University School of Medicine (SH9H-2021-A744-SB).

### Bioinformatics analysis

Three bulk RNA expression datasets (GSE119195, GSE132714, GSE172357) and one scRNA-seq dataset were obtained from the GEO database and from our previously published study [22, 24–26]. Single-cell transcriptomic data were analyzed in R (version 4.4.3) using the Seurat package. Low-quality cells were excluded based on thresholds for gene counts, unique molecular identifier counts, and mitochondrial gene content. Data were log-normalized, and batch effects were corrected using the Harmony algorithm. Principal component analysis was performed, and the leading principal components were used to generate uniform manifold approximation and projection (UMAP) plots for visualization and clustering. DEGs were identified using the FindMarkers function in Seurat for single-cell data and the limma package for bulk RNA expression data, with significance defined as *P* value < 0.05. Significantly altered DEGs were subjected to GO enrichment analysis using the Metascape online platform [46], and signaling network diagrams were constructed. GSEA was performed with the clusterProfiler package in R. For GSEA, genes were ranked by log₂ fold change and analyzed against the MSigDB reference gene sets, with enrichment considered significant at a false discovery rate < 0.25. The stromalization degree of each sample was assessed using the ESTIMATE algorithm to calculate stromal scores from the GEO-derived expression matrices.

### Statistical analysis

Data are presented as the mean ± standard deviation (SD) from a minimum of three independent experiments. Comparisons between two groups were performed using a two-tailed Student’s t-test. Changes in prostate volume and associated symptoms before and after treatment were analyzed using the Wilcoxon paired test. Correlations between variables were evaluated by linear regression analysis, with the coefficient of determination (R^2^) used to indicate the goodness of fit. All statistical analyses were conducted using SPSS software (version 23.0), and a *P* value < 0.05 was considered statistically significant.

## Supplementary Information

Supplementary Material 1.

## Data Availability

The data and materials that support the findings of this study are available from the corresponding author B.X. upon reasonable request.

## Abbreviations

BPH: Benign prostatic hyperplasia
LUTS: Lower urinary tract symptoms
TURP: Transurethral resection of the prostate
RTK: Receptor tyrosine kinase
RTKi: RTK inhibitor
CSF1R: Colony-stimulating factor 1 receptor
GEO: Gene Expression Omnibus
GSEA: Gene set enrichment analysis
scRNA-seq: Single-cell RNA sequencing
HPA: Human Protein Atlas
IHC: Immunohistochemistry
ELISA: Enzyme-linked immunosorbent assay
ccRCC: Clear cell renal cell carcinoma
CT: Computed tomography
IPSS: International Prostate Symptom Score
PVR: Post-void residual urine volume
Qmax: Maximum urinary flow rate
UMAP: Uniform manifold approximation and projection
SD: Standard deviation
NES: Normalized enrichment score
