# Supplementary material for: Fibroblasts promote the progression of benign prostatic hyperplasia through colony-stimulating factor 1 receptor-mediated RTK signaling in prostatic epithelial cells
Source: Mol Biomed. 2025 Nov 28;6:126. doi: 10.1186/s43556-025-00360-w (PMC12662972; doi:10.1186/s43556-025-00360-w)

Fibroblasts Promote the Progression of Benign Prostatic Hyperplasia through Colony-Stimulating Factor 1 Receptor-Mediated RTK Signaling in Prostatic Epithelial Cells

Ming Zhan[1,2#], Ruifeng Yang[3#], Yue Gu[1,4#], Jun Zhu[1#], Miaomiao Guo[5#], George Pupwe[6], Xiaohua Huang[2], Huan Xu[1], Zhilian Jia[2], Kyle Takehiro[7], Chong Liu[1], Bingyu Li[8], Yiwei Wang[1], Yanbo Chen[1*], Xianjin Wang[9*], Qi Chen[1*], Bin Xu[1*]

[1]Department of Urology, Shanghai Ninth People's Hospital, Shanghai Jiao Tong University School of Medicine, Shanghai 200011, China

[2]Department of Systems Biology, Beckman Research Institute, City of Hope, Monrovia, CA 91016, USA

[3]Department of Urology, Fudan University Shanghai Cancer Center, Shanghai 200032, China

[4]Department of Urology, Putuo People's Hospital, Tongji University School of Medicine, Shanghai 200060, China.

[5]Department of Molecular Diagnostics & Endocrinology, Shanghai Ninth People's Hospital, Shanghai Jiao Tong University School of Medicine, Shanghai 200011, China.

[6]Department of Pathology, City of Hope, Duarte, CA 91010, USA

[7]Arcadia High School, Arcadia, CA 91006, USA

[8]Department of Pathology, Mount Sinai West/Morningside Hospitals, New York, NY 10025, USA.

[9]Department of Urology, Ruijin Hospital, Shanghai Jiao Tong University School of Medicine, Shanghai 200025, China.

[#]These authors contributed equally to this work.

[*]Corresponding Authors: Bin Xu (chxb2004@shsmu.edu.cn), Qi Chen (qiqi_chenqi@163.com), Xianjin Wang (xianjin09@163.com), Yanbo Chen

(fantasy_cyb@163.com)

Supplementary Methods

Cell culture and treatments

The BPH-1 cell line was obtained from the Shanghai Institutes for Biological Sciences. HEK-293T and RWPE-1 cell lines were purchased from the American Type Culture Collection. RWPE-1 cells were cultured in K-SFM (Gibco, USA) supplemented with 0.05 mg/mL bovine pituitary extract (Gibco) and 5 ng/mL human recombinant EGF (Gibco). BPH-1 cells were maintained in RPMI-1640 medium (Gibco), and HEK-293T cells were cultured in DMEM (Gibco); both media were supplemented with 10% FBS (Gibco). Primary prostate luminal epithelial cells were isolated from BPH or normal prostate tissues. Tissue specimens were minced, digested with 5 mg/mL collagenase II (Thermo Fisher Scientific, USA) for 2h at 37 °C, and subjected to differential adhesion to remove rapidly adherent stromal cells. The remaining epithelial-enriched fraction was further dissociated with TrypLE Express (Gibco) containing Y-27632 (Sigma-Aldrich, USA), filtered through a 40 μm strainer, and cultured in K-SFM supplemented with BPE, EGF, dihydrotestosterone (Sigma-Aldrich), and Y-27632 to support luminal cell viability and phenotype maintenance. All cultures were maintained at 37 °C in a humidified atmosphere containing 5% CO_2, with regular mycoplasma testing to ensure culture integrity. Sunitinib (Selleck, USA), pexidartinib (Selleck, USA), human IL34 (R&D Systems, USA), and CSF1 (MedChemExpress, USA) recombinant proteins were used to treat cells. Recombinant human IL34 and CSF1 protein was reconstituted in sterile 1× PBS (pH 7.4) containing 0.1% endotoxin-free recombinant human serum albumin. Treatment concentrations were as follows: sunitinib, 1 or 2 μM; pexidartinib, 5 μM, IL34, 50 ng/mL; CSF1, 50 ng/mL. Cells treated with the corresponding solvent (DMSO or PBS) served as controls.

MTT and colony formation assays

BPH-1 cells, RWPE-1 cells, and primary prostate luminal cells derived from BPH or normal prostate tissues were transfected or treated with the indicated drugs, then seeded at a density of 3,000 cells per well in 96-well plates and incubated at 37 °C for 0–60 h

or 0–5 days. Cell proliferation was assessed using the MTT assay (Sigma-Aldrich). Absorbance was measured at 490 nm with a Synergy 2 microplate reader (BioTek, USA). For colony formation assays, prostate cells were plated in 6-well plates at a density of 200 cells per well and cultured for two weeks until visible colonies formed. Colonies were fixed with 4% paraformaldehyde, stained with 0.1% crystal violet, and counted under a light microscope.

BrdU labeling

BPH-1 cells treated with 2 μM sunitinib or an equivalent volume of DMSO were incubated with 10 μM BrdU (Sigma-Aldrich) for 10 h. Cells were fixed in PBS containing 4% formaldehyde for 10 min at room temperature and permeabilized with 0.5% Triton X-100 for 10 min. Following incubation with an anti-BrdU antibody (Proteintech, 66241-1-Ig, 1:500, China) for 30 min, nuclei were counterstained with DAPI (Beyotime, China) for 5 min. BrdU-positive cells were visualized using a fluorescence microscope and quantified with the Analyze Particles function in ImageJ software.

Flow cytometry for apoptosis detection

Apoptosis was assessed by flow cytometry using Annexin V–propidium iodide (PI) staining with an Annexin V–FITC Apoptosis Detection Kit (Sigma-Aldrich), according to the manufacturer's protocol. Briefly, cells treated with 2 μM sunitinib or an equivalent volume of DMSO for 36 h were washed, resuspended in 100 μL staining solution, and incubated for 10 min in the dark. Samples were analyzed on a BD Accuri C6 Flow Cytometer (BD Biosciences, USA).

RNA extraction and qPCR assays

BPH and normal prostate tissues were immediately flash-frozen in liquid nitrogen and homogenized in TRIzol reagent (Invitrogen, USA) for RNA extraction. Cultured cells were lysed directly in TRIzol for RNA isolation. cDNA was synthesized using the PrimeScript RT Reagent Kit (Takara Bio, China) according to the manufacturer's instructions. qPCR was performed on an Applied Biosystems ViiA™ 7 Real-Time PCR

System using SYBR Premix Ex Taq (Takara Bio). mRNA expression levels were normalized to β-actin, and relative expression was calculated using the $2^{-\Delta\Delta CT}$ method. All primers were synthesized by Sangon Biotech (China), and their sequences are provided in Supplementary Table 1.

ELISA

The concentrations of human IL34 and CSF1 in BPH tissue lysates and cell culture supernatants were determined using a sandwich ELISA performed on Nunc MaxiSorp 96-well plates (Sigma-Aldrich). Capture and detection antibodies specific for IL34 and CSF1, as well as the streptavidin–HRP conjugate, were obtained from Sigma-Aldrich. The substrate solution containing o-phenylenediamine dihydrochloride (Sigma-Aldrich) was prepared according to the manufacturer's instructions. Following color development, absorbance was measured at 450 nm using a BioTek microplate reader, and cytokine concentrations were calculated from standard curves generated in parallel.

Immunoblot analysis

Total protein was extracted from cells using RIPA lysis buffer (Beyotime) supplemented with a protease inhibitor cocktail (Sigma-Aldrich). Protein concentrations were determined using the BCA Protein Assay Kit (Thermo Fisher Scientific) according to the manufacturer's instructions. Equal amounts of protein were separated by SDS–polyacrylamide gel electrophoresis and transferred to polyvinylidene difluoride membranes (Millipore, USA). Membranes were blocked with 5% non-fat milk in TBST and incubated overnight at 4 °C with the following primary antibodies (all from Cell Signaling Technology): phospho-mTOR (#5536, 1:800), mTOR (#2972, 1:1000), phospho-AKT (#4060, 1:2000), AKT (#9272, 1:1000), CSF1R (#3152, 1:800), and β-actin (#4967, 1:2000). After washing, membranes were incubated for 1 h at room temperature with HRP-conjugated secondary antibodies (Cell Signaling Technology, USA). Immunoreactive bands were detected using an enhanced chemiluminescence system (ChemiDoc Imaging System, Bio-Rad, USA), and band intensities were quantified with Image Lab software (Bio-Rad).

IHC staining

Formalin-fixed, paraffin-embedded mouse prostate tissues, together with BPH and normal human prostate specimens, were sectioned and mounted onto charged slides. Sections were deparaffinized, rehydrated, and treated to block endogenous peroxidase activity. Ki-67 staining was performed using an anti-Ki-67 antibody (Cell Signaling Technology, #9449; 1:600 dilution) following a standard immunoperoxidase protocol. The proportion of Ki-67–positive cells was determined by counting 500 epithelial cells per mouse prostate sample. CSF1R staining was performed using the same procedure with an anti-CSF1R antibody (Sigma-Aldrich, CAB008970; 1:500 dilution). Staining was evaluated semi-quantitatively by scoring staining intensity (I: 0 = negative, 1 = weak, 2 = moderate, 3 = strong) and the percentage of positive cells (P: 0–5% = 0, 6–35% = 1, 36–70% = 2, >70% = 3). The final immunoreactivity score (Q) was obtained by multiplying I and P, with scores ≥ 4 considered high expression. All assessments were conducted independently by two experienced pathologists in a double-blind manner to ensure unbiased results.

Supplementary Table 1. Baseline clinical characteristics of ccRCC patients treated with sunitinib

Characteristics	ccRCC patients		
	Cohort 1 (SNP)	Cohort 2 (RJ)	Cohort 3 (SCC)
Number of patients	16	39	27
Median age at sunitinib initiation, years (range)	63 (52-78)	65 (55-76)	62 (56-80)
Median body weight, kg (range)	65 (50-83)	66 (52-81)	67 (53-85)
IMDC risk group, n (%)			
Favorable	3 (18.8)	11 (28.2)	6 (22.2)
Intermediate	8 (50.0)	16 (41.0)	12 (44.4)
Poor	5 (31.3)	12 (30.8)	9 (33.3)
Median exposure time to sunitinib, days (range)	202 (92-454)	227 (112-519)	196 (126-403)
Baseline prostate volume, n (%)			
Mild	4 (25.0)	11 (28.2)	8 (29.6)
Moderate	10 (62.5)	25 (64.1)	16 (59.3)
Severe	2 (12.5)	3 (7.7)	3 (11.1)
Anemia, n (%)			
Grades 1–2	5 (31.3)	12 (30.8)	8 (29.6)
Grades 3–4	0 (0.0)	1 (2.6)	1 (3.7)
Grades 5	0 (0.0)	0 (0.0)	0 (0.0)
Hypertension, n (%)			
Grades 1–2	4 (25.0)	9 (23.1)	7 (25.9)
Grades 3–4	2 (12.5)	5 (12.8)	4 (14.8)
Grades 5	0 (0.0)	0 (0.0)	0 (0.0)
Lymphopenia, n (%)			
Grades 1–2	3 (18.8)	8 (20.5)	5 (18.5)
Grades 3–4	0 (0.0)	0 (0.0)	0 (0.0)
Grades 5	0 (0.0)	0 (0.0)	0 (0.0)
Thrombopenia, n (%)			
Grades 1–2	5 (31.3)	14 (35.9)	8 (29.6)
Grades 3–4	1 (6.3)	2 (5.1)	1 (3.7)
Grades 5	0 (0.0)	0 (0.0)	0 (0.0)

IMDC: International metastatic renal-cell carcinoma database consortium.

Adverse events were defined using the National Cancer Institute Common Terminology Criteria for Adverse Events (version 4.0).

Supplementary Table 2. Primers sequences for qPCR.

Gene	Primer Sequences	
FLT1	Forward (5'-3')	GAACTGAGTTTAAAAGGCACCCA
	Reverse (5'-3')	TGTTTGCCATTTCTTCCACAGG
KDR	Forward (5'-3')	TGTTTCTCTTGATCTGCCCAGG
	Reverse (5'-3')	ACTCAGTCACCTCCACCCTT
FLT4	Forward (5'-3')	CCTGCGACTGTGGCTCTG
	Reverse (5'-3')	GTCCTCGCTGTCCTTGTCTC
PDGFRA	Forward (5'-3')	ACAACCACACTCAGACAGAAGA
	Reverse (5'-3')	AGTCTCGGGATCAGTTGTGC
PDGFRB	Forward (5'-3')	CAGTGGGGAACAGACAGTCC
	Reverse (5'-3')	CTCCTCCTCCCAGTACGTCA
KIT	Forward (5'-3')	TCTGACGTCAATGCTGCCAT
	Reverse (5'-3')	GGCAGTACAGAAGCAGAGCA
FLT3	Forward (5'-3')	TCTCTGGGTCTTTAAGCACAGC
	Reverse (5'-3')	GATATGCAGACCAGGGCGTC
RET	Forward (5'-3')	GCAACGGCCTTCCATCTGAA
	Reverse (5'-3')	TGCAGGCCCCATACAATTTGA
CSF1R	Forward (5'-3')	GACAGGAGAGAGCGGGACTA
	Reverse (5'-3')	AGCAGGTCAGGTGCTCACTA
CSF1	Forward (5'-3')	TTGTTGGTCTGTCTCCTGGC
	Reverse (5'-3')	ATCTCTGAAGCGCATGGTGT
IL34	Forward (5'-3')	CAGGACGTGCTGCTCGAG
	Reverse (5'-3')	GCAGTACAGCAGCTCCATGA
GAPDH	Forward (5'-3')	GAAGGGCTCATGACCACAGT
	Reverse (5'-3')	GGATGCAGGGATGATGTTCT
**shRNA**		
sh-Con	5′-UUCUCCGAACGUGUCACGUTT-3′	
sh-CSF1R	5′-GGGACUUUGUCCUGUUCAACU-3′	

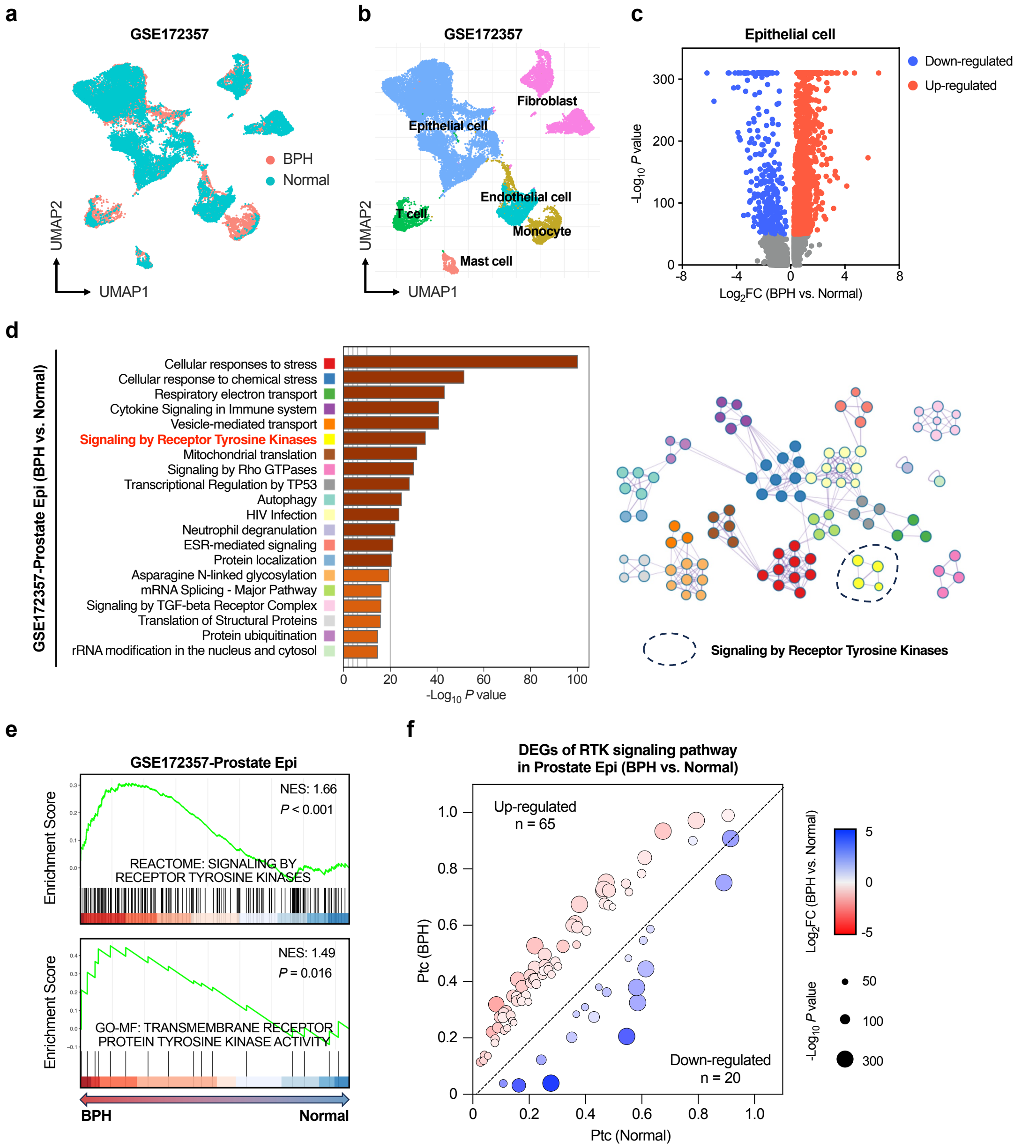

Supplementary Fig. 1 Single-cell transcriptomic analysis reveals activation of RTK signaling in prostatic epithelial cells from BPH tissues. **a–b** UMAP visualization of scRNA-seq data (GSE172357) from prostate tissues, with cells colored according to sample group (BPH and normal) (**a**) or by major cell populations (**b**) identified based on canonical marker gene expression. **c** Volcano plot of DEGs between BPH and normal epithelial cells, with upregulated (red) and downregulated (blue) genes highlighted. **d** GO and Reactome pathway enrichment analysis of DEGs in epithelial cells from BPH versus normal prostates highlighting significant enrichment of RTK signaling-related terms. **e** GSEA showing activation of RTK signaling in BPH epithelial cells (NES and

P values indicated). **f** Percentage of epithelial cells (Pct) from BPH and normal prostate tissues expressing DEGs related to the RTK signaling pathway.

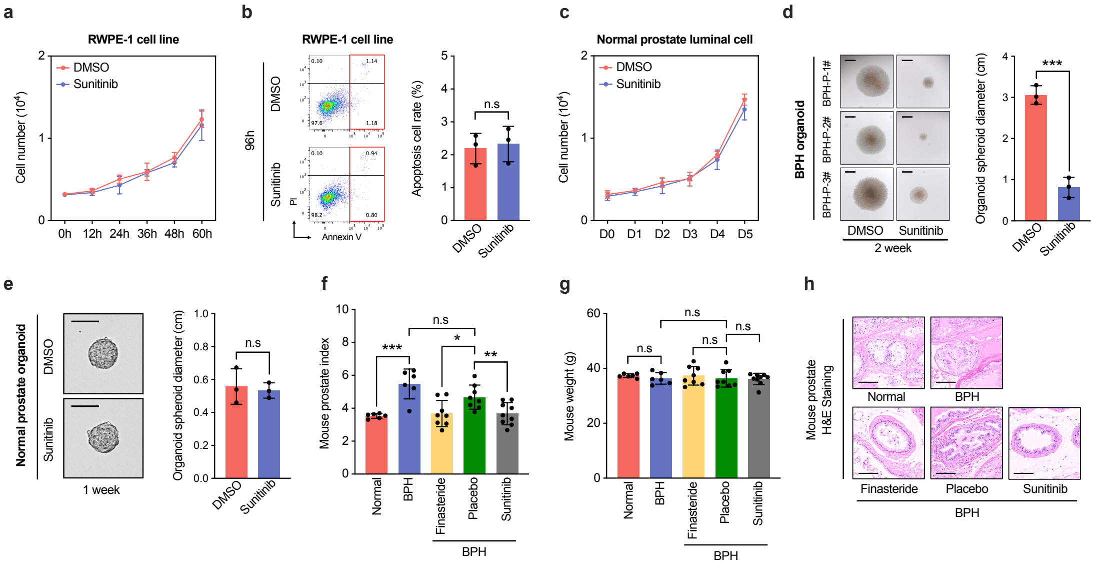

Supplementary Fig. 2 Sunitinib does not significantly affect proliferation or viability of normal prostate epithelial cells. **a** Growth curves of RWPE-1 cells treated with sunitinib or DMSO for up to 60 h, assessed by MTT assay. **b** Flow cytometric quantification of apoptosis in RWPE-1 cells after 96 h treatment with sunitinib or DMSO. **c** Growth curves of primary luminal epithelial cells from normal prostate tissues treated with sunitinib or DMSO for up to 5 days, assessed by MTT assay. **d** Representative images and spheroid diameter quantification of BPH-derived prostate organoids cultured for 2 weeks with sunitinib or DMSO. Scale bars: 1.0 cm. **e** Representative images and spheroid diameter quantification of Normal-derived prostate organoids cultured for 1 week with sunitinib or DMSO. Scale bars: 1.0 cm. **f–g** Prostate index (**e**) and body weight (**f**) in normal and testosterone-induced BPH mice treated with placebo, finasteride, or sunitinib for 8 weeks. **h** Representative H&E staining of prostate tissues from the indicated mouse groups. Scale bar: 100 µm. Data are presented as mean ± SD from at least three independent experiments or biological replicates. Statistical significance was determined by Student's *t*-test; *$P < 0.05$, **$P < 0.01$, ***$P < 0.001$; n.s, not significant.

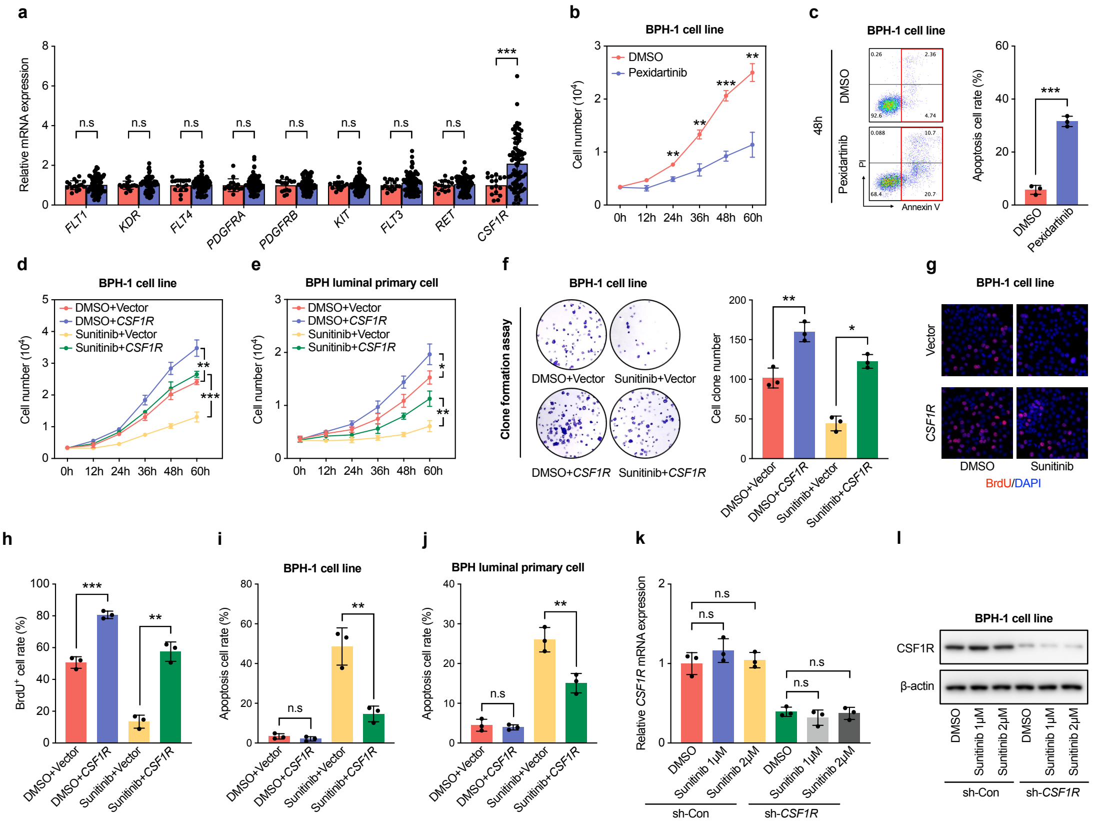

Supplementary Fig. 3 CSF1R overexpression rescues the inhibitory effects of sunitinib on proliferation and clonogenicity in BPH epithelial cells. **a** Quantification of mRNA expression for nine established sunitinib target genes in an independent validation cohort using qPCR (normal prostate, n = 16; BPH, n = 80). **b** Growth curves of BPH-1 cells treated with pexidartinib or DMSO for up to 60 h, measured by MTT assay. **c** Flow cytometric analysis of apoptosis in BPH-1 cells following 48 h treatment with pexidartinib, assessed by Annexin V/PI staining. **d-e** Growth curves of BPH-1 cells **(d)** and primary luminal epithelial cells from BPH tissues **(e)** transfected with control vector or CSF1R overexpression plasmid, treated with DMSO or sunitinib. **f** Colony formation assays of BPH-1 cells transfected with control vector or CSF1R overexpression plasmid, treated with DMSO or sunitinib. **g-h** BrdU incorporation assays showing proliferative activity in BPH-1 cells transfected with control vector or CSF1R overexpression plasmid, treated with DMSO or sunitinib. **i–j** BPH-1 cells **(i)** and primary luminal epithelial cells from BPH tissues **(j)** transfected with control vector or CSF1R overexpression plasmid, treated with DMSO or sunitinib. **k–l** qPCR (**k**) and

Western blotting (**I**) analysis of CSF1R mRNA and protein expression in BPH-1 cells (sh-Con and sh-CSF1R) treated with sunitinib or DMSO. Data are presented as mean ± SD from at least three independent experiments or biological replicates. Statistical significance was determined by Student's *t*-test; *$P < 0.05$, **$P < 0.01$, ***$P < 0.001$; n.s, not significant.

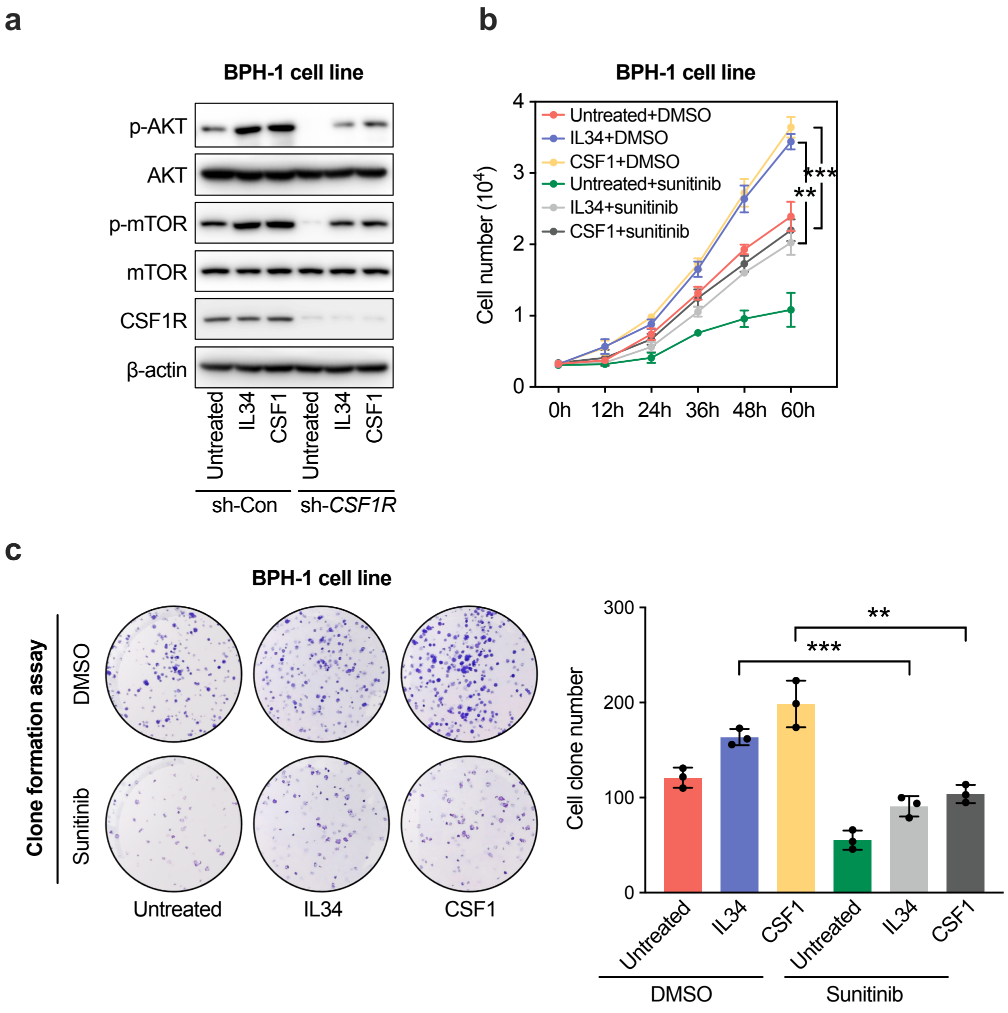

Supplementary Fig. 4 Sunitinib inhibits CSF1- and IL34-induced proliferation and clonogenicity in BPH epithelial cells through blockade of CSF1R signaling. **a** Western blot analysis of CSF1R, phosphorylated AKT (p-AKT), total AKT, phosphorylated mTOR (p-mTOR), and total mTOR in BPH-1 cells transfected with treated with CSF1 or IL34 after transfection with CSF1R shRNA or control shRNA. β-actin served as a loading control. **b–c** MTT **(b)** and colony formation assays **(c)** of BPH-1 cells treated with CSF1or IL34 in the presence or absence of sunitinib. Data are presented as mean ± SD from at least three independent experiments. Statistical significance was determined by Student's *t*-test; $^{**}P < 0.01$, $^{***}P < 0.001$.

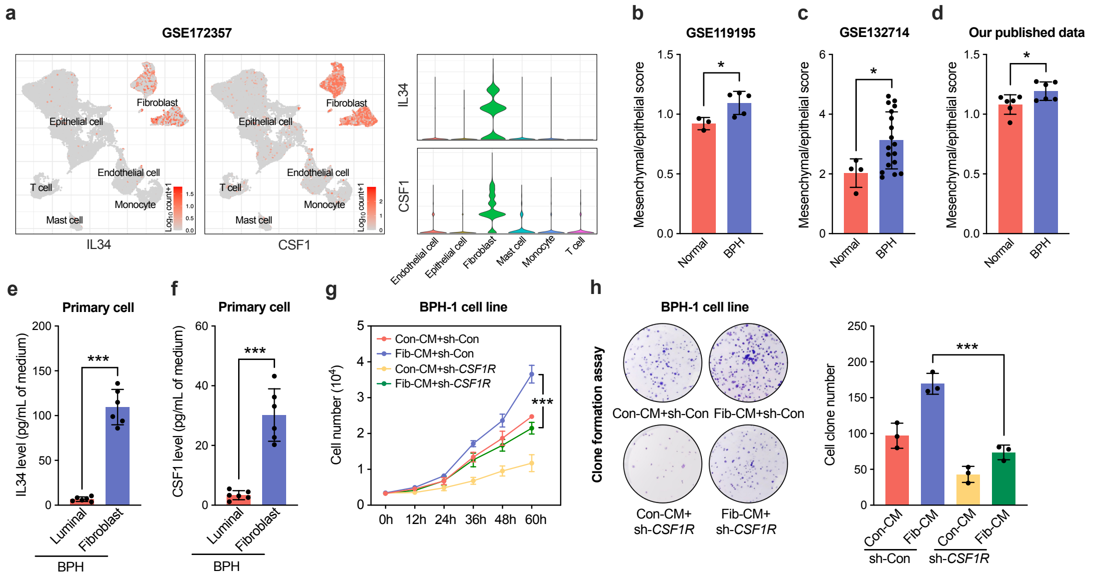

Supplementary Fig. 5 Fibroblast-derived CSF1 and IL34 promote BPH epithelial proliferation via CSF1R signaling. **a** scRNA-seq analysis (GSE172357) showing cell-type–specific expression of CSF1 and IL34 in prostate tissue. **b–d** Mesenchymal/epithelial score comparison in normal versus BPH tissues from the GSE119195 **(b)**, GSE132714 **(c)**, and from our previously published transcriptomic dataset **(d)**. **e–f** ELISA quantification of CSF1 (**e**) and IL34 (**f**) levels in culture supernatants from fibroblasts and luminal epithelial cells isolated from BPH tissues. **g–h** Growth curves (**g**) and colony formation assays (**h**) of BPH-1 cells cultured with control medium (Con-CM) or fibroblast-conditioned medium (Fib-CM) after transfection with control shRNA (sh-Con) or CSF1R shRNA (sh-CSF1R). Data are presented as mean ± SD from at least three independent experiments. *$P < 0.05$, ***$P < 0.001$.

Uncropped blots

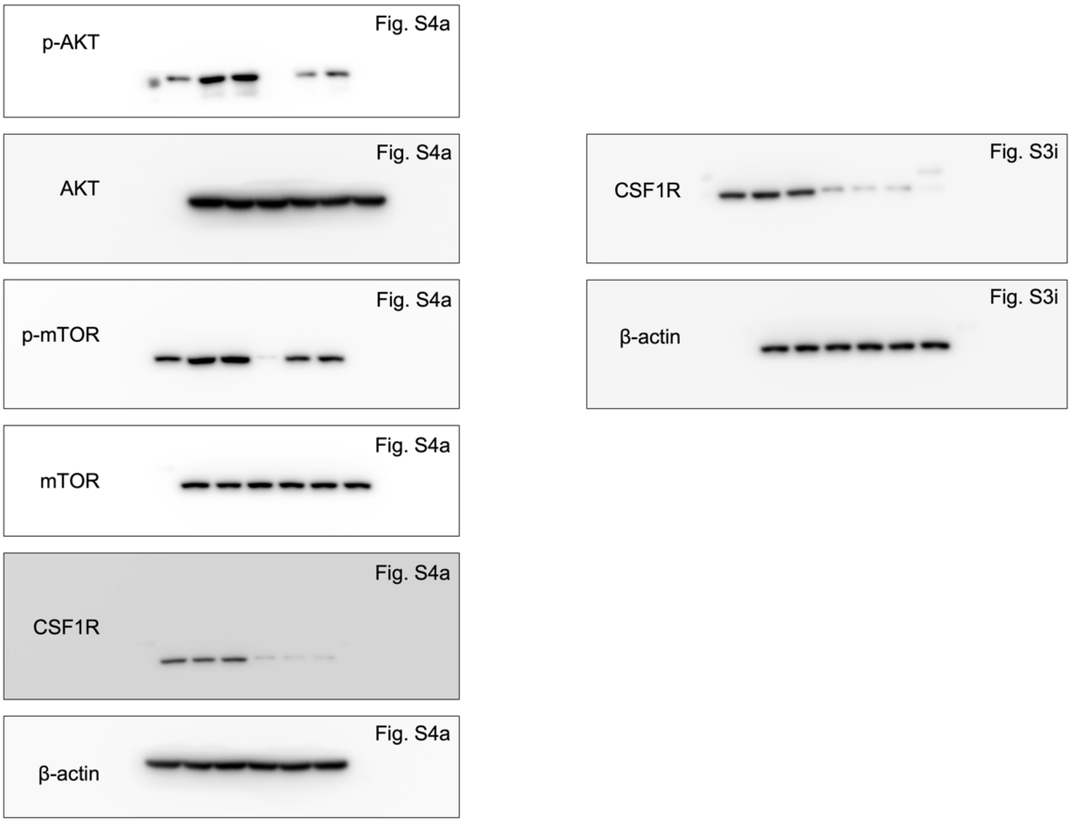

Supplement: Supplementary file 1 — Supplementary Material 1. [file 43556_2025_360_MOESM1_ESM.pdf]
